# Tele-ophthalmology as an effective triaging tool for acute ophthalmic concerns

**DOI:** 10.3389/fopht.2024.1511378

**Published:** 2025-01-14

**Authors:** Natalie A. Townsend, Shalini Shah, Joshua Reyes, Justin H. Townsend, Alison Bozung, Giselle Ricur, Rami J. Aboumourad

**Affiliations:** Bascom Palmer Eye Institute, University of Miami, Miami, FL, United States

**Keywords:** telehealth, telemedicine, tele-ophthalmology, ophthalmology, optometry

## Abstract

**Introduction:**

The purpose of this study is to determine baseline demographics and utilization trend of an on-demand, synchronous tele-ophthalmology triage program in evaluating acute ophthalmic concerns during the COVID-19 Public Health Emergency.

**Methods:**

Setting: Single-center retrospective chart review of telemedicine visits conducted by ophthalmologists and optometrists from University of Miami’s Bascom Palmer Eye Institute. Patient population: 6227 patients comprised 7138 telehealth encounters. All patient encounters were included in the retrospective review without exclusions and only the primary diagnoses were categorized from October 1, 2020 to April 30, 2023. Main outcomes measures: Descriptive statistics of the telemedicine model, utilization trends, baseline patient demographics, and primary diagnoses were performed for all virtual eye care encounters during the study period.

**Results:**

Utilization of the synchronous telemedicine platform increased during the study period. The median age of patients was 51 (IQR, 36-65) years. Patients predominantly self-identified as female (63.27%), White (72.7%), and non-Hispanic/Latino (48.2%). General external adnexa (44.1%), conjunctival disorders (15.5%) and ocular surface symptoms (15.4%), made up 75.0% of the visits during the study period. Furthermore, 63.4% of patients were new to Bascom Palmer Eye Institute, 67.1% had never engaged in telemedicine, and 96.5% of encounters were successfully completed through video conferencing.

**Discussion:**

During the COVID-19 pandemic, there was significant utilization of an on-demand synchronous ocular telemedicine program to address acute concerns. This retrospective chart review demonstrates the utility of telemedicine as an important and effective tool to triage and provide care during the COVID-19 Public Health Emergency.

## 1 Introduction

The coronavirus disease 2019 (COVID-19) pandemic incited significant changes in the way healthcare is delivered. The Department of Health and Human Services (HHS) declared a federal Public Health Emergency (PHE) for COVID-19, under Section 319 of the Public Health Service Act, on January 31, 2020 and it expired on May 11, 2023. During this time period extensive efforts were taken to improve the quality of and access to telemedicine (1). Telemedicine allowed healthcare providers to interact with patients when entities limited in-person exams, and patients were reluctant to enter hospitals/clinics due to concern of disease exposure (2, 3).

Multiple prior studies by the American Medical Association have established widespread support and increased use of telehealth, specifically synchronous telemedicine, by physicians during the pandemic (4, 5). Telemedicine utilization increased significantly in 2020 compared to 2018, and was greatest in medical specialties such as nephrology, neurology, hematology, and oncology (4–6). Telemedicine provided patients access to healthcare providers even during lockdown measures. A single-center review of telehealth outcomes by multidisciplinary medical subspecialties demonstrated a clinical diagnostic accuracy of 86.9% for virtual exams compared to in-person exams; 91.7% concordance for tele-ophthalmology (7).

Traditionally, ophthalmologic exams have been preferred to be conducted via in-person examination due to unique, specific instrumentation used during evaluation to gather data such as visual acuity, pupillometry, ocular motility, assessment of peripheral visual field, measurement of the intraocular pressure, and examination of the anterior and posterior segments using a slit lamp biomicroscope and indirect ophthalmoscope. Ophthalmology, however, was among many fields of medicine that sought to create or expand telehealth services (8). Models used in tele-ophthalmology care include synchronous care (video conferencing), asynchronous care (store-and-forward), remote patient monitoring, and mobile health (1, 9, 10). Despite ophthalmology having the most drastic patient volume decline, estimated at 81% during the initial pandemic, compared to other fields, teleconsultation utilization in ophthalmology was low during the pandemic and was conducted primarily in an asynchronous fashion (2, 6, 11, 12). At present, ophthalmology does not have well-validated devices to provide this data for remote patients, but there are several devices seeking to obtain these measurements outside the traditional eye exam room (1, 10).

In April 2020, the Rapid Virtual Eye Care^®^ program at the University of Miami Bascom Palmer Eye Institute (BPEI) was initiated to provide on-demand, synchronous ophthalmologic video or audio-only conferencing to patients with acute eye concerns. As a novel virtual triage program, this allowed symptomatic patients or those referred for ophthalmologic care to seek consultation quickly and from their home. The adoption and continuation of synchronous video conferencing exams in ophthalmology has been limited, and utilization has not been well-described in the literature (3, 9, 13). In this study, we report a proof-of-principle model and utilization characteristics of a large synchronous tele-ophthalmology program conducted at an academic center during the COVID-19 PHE, validating the role of telehealth in providing access to care to patients and triaging acute ophthalmic concerns.

## 2 Materials and methods

### 2.1 Clinic characteristics

This tele-ophthalmology program provided on-demand patient-to-doctor live consultation eight hours per day, six days per week, with appointments every 20 minutes. A specific visit type to facilitate scheduling was established in the electronic medical record (EMR) system, Epic (Epic; Epic System Corporation, Wisconsin, USA), used by the University of Miami.

On-demand care allowed patients to be scheduled for an appointment within three consecutive days prior and up to the time of the appointment requested. A scheduling agent could make the appointment via phone, or the patient could schedule themself via a self-scheduling feature on the eye institute’s website (www.rapideyecare.com).

Once scheduled, patients accessed the virtual appointment via the university-based portal, MyUHealthChart (MyChart; Epic Systems Corporation, Wisconsin, USA), system from a smartphone, tablet, or computer. Patients signed consents to be seen via telehealth and were provided an electronic video conferencing link to connect for the visit.

Virtual clinic coordinators would ensure patients could access their MyUHealthChart portal and connect via Zoom (Zoom Video Communications, Qumu Corporation, California, USA) for a video conference, the preferred method by providers and University telehealth guidelines. If the patient was unable to connect via Zoom, patients were contacted via Doximity Dialer or Doximity Video (Doximity Inc., California, USA) and verbal consent was obtained and documented in the chart. There was a designated coordinator for the service whose role was to collect medical, surgical, and social histories, as well as chief complaint and history of present illness.

During the study period, fifteen state-licensed ophthalmologists or optometrists rotated on the service and would connect to the visit after the coordinator. The eye care provider would review the chief complaint and perform a gross evaluation of the patient’s external adnexa and anterior segment via the patient’s device camera, when applicable.

At the completion of the visit, the provider would document the primary diagnoses using *Codes from the International Classification of Disease, Tenth Revision, Clinical Modifications (ICD-10)*, the treatment recommendations, level of service, and follow-up appointment details as indicated.

### 2.2 Study design and data collection

This study is a single-center, retrospective, cross-sectional chart review of BPEI’s Rapid Virtual Eye Care^®^ telemedicine encounters from October 1, 2020, through April 30, 2023. BPEI is affiliated with the UHealth System of the University of Miami, Miami, FL. This study was conducted in accordance with guidelines from the Declaration of Helsinki and was approved by the Institutional Review Board at the University of Miami.

All patients who sought eye care under the program during the study duration were included; no exclusion criteria applied.

### 2.3 Outcomes

Primary outcomes of the study included patient utilization, demographics, and primary diagnosis from the visit. Patient demographics including age, and self-reported gender and race/ethnicity were analyzed. ICD-10 codes were categorized into groups of diagnoses which can be found separately in the annexes. For patients with multiple diagnoses, manual chart review was conducted to confirm which diagnosis was the primary concern addressed in the visit. Manual chart review was completed by AB and RA, two of the providers in the Rapid Virtual Eye Care^®^ service.

Secondary outcomes included patient health insurance status, whether they were new or established patients, and teleconference model specifics (video or audio-only). Established patients were considered to have had care within the institute, from the same specialty, within the 3 years preceding the telehealth appointment.

### 2.4 Statistical analyses

Analyses were performed using IBM SPSS Statistics (Version 28). The One-Sample Kolmogorov-Smirnov normality test and histogram plot were utilized for the continuous variable, age at visit, to determine if the distribution was normal. The non-normally distributed results are reported as median (interquartile range [IQR]). Descriptive statistics for categorical variables were reported as count and percentages.

Statistical significance of differences between the continuous variable was assessed via the Kruskal-Wallis test, while categorical variables were assessed via the Pearson χ^2^ test. Significance level (α) was set to 0.05. Adjustments for multiple analyses or outcomes were not performed.

## 3 Results

### 3.1 Patient utilization and demographics

**Figure 1:**
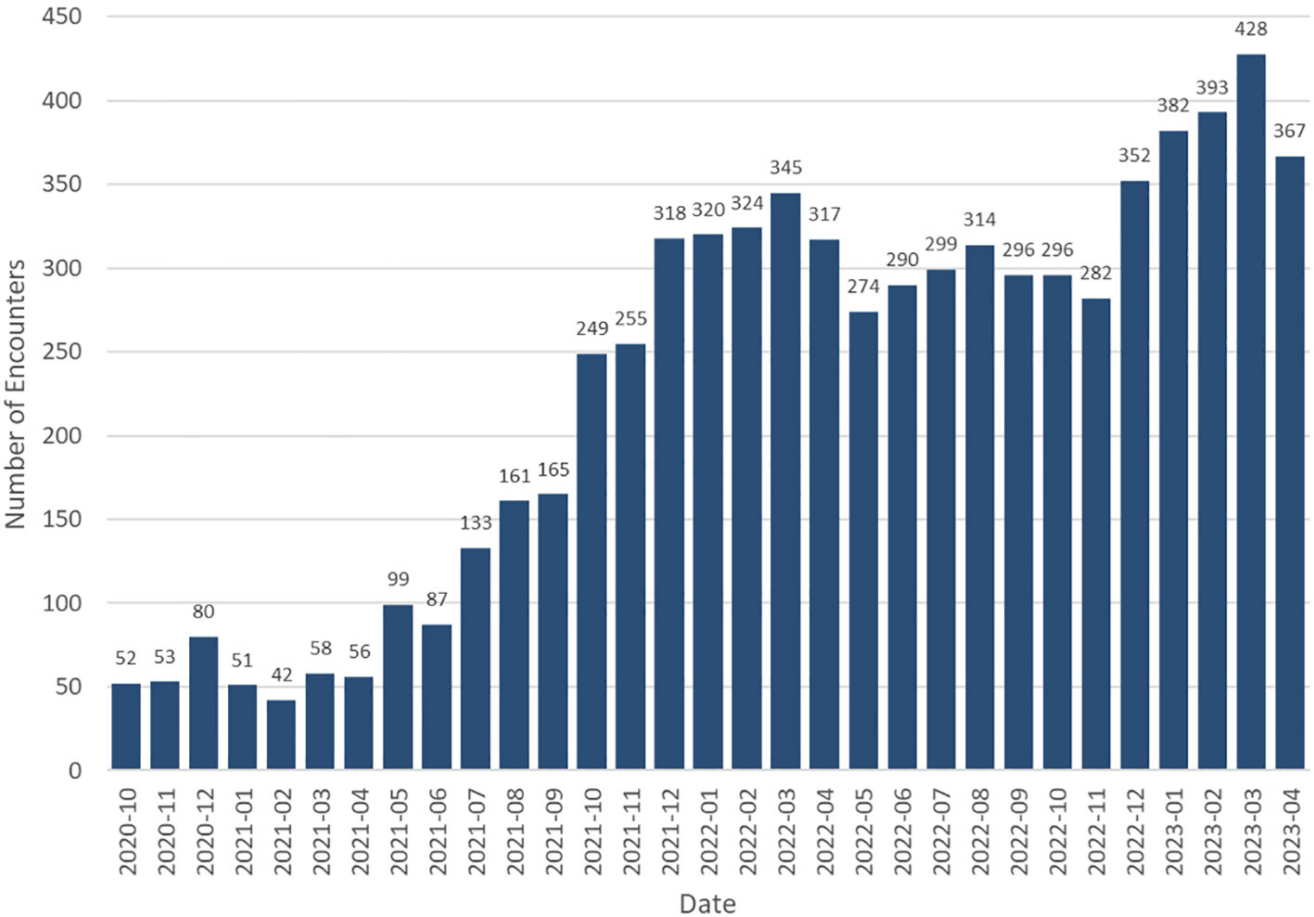
Number of encounters seen through Rapid Virtual Eye Care^®^ per month.

**Figure 2:**
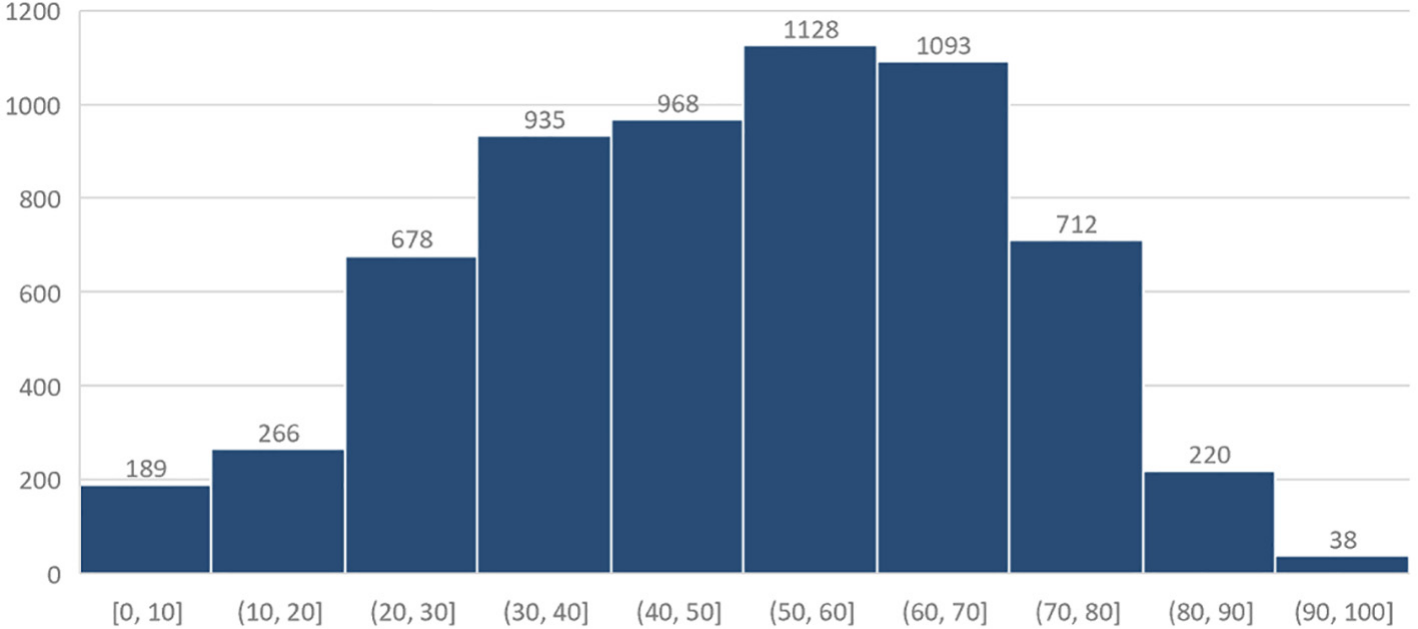
Histogram of age.

From October 1, 2020, through April 30, 2023, 6227 patients completed 7138 virtual ophthalmologic encounters. Monthly volume analysis shows increased utilization during the duration of the study (**Figure 1**). The median age of patients was 51 (interquartile range, 36-65) years, and 3964 (63.7%) identified as female (**Figure 2**, **Table 1**). Patients self-identified their race as follows: 4526 (72.7%) White, 582 (9.3%) Black or African American, 118 (1.9%) Asian, 13 (0.2%) American Indian or Alaska native, 9 (0.1%) native Hawaiian or other Pacific Islander, and 979 (15.7%) were unknown or chose not to answer. Regarding ethnicity, 3000 (48.2%) of patients identified as Non-Hispanic or Latino, followed by 2297 (36.9%) Hispanic or Latino, and 930 (14.9%) were unknown or chose not to answer.

**Table 1:** Demographics and characteristics of patients seen through Rapid Virtual Eye Care^®^.

	Total^a^	Female	Male	P^b^
**Gender**	6227	3964 (63.7%)	2263 (36.3%)	–
**Age, median (IQR), y**	51 (35-65)	52 (36-65)	50 (34-65)	.14 ^c^
Age range				
**0-18**	335 (5.4%)	175 (2.8%)	160 (2.6%)	<.001
**19-40**	1727 (27.8%)	1104 (17.7%)	623 (10.0%)	.81
**41-60**	2096 (33.7%)	1364 (21.9%)	732 (11.8%)	.10
**61+**	2063 (33.2%)	1319 (21.2%)	744 (12.0%)	.77
Race				
**American Indian or Alaska Native**	13 (0.2%)	9 (0.1%)	4 (0.1%)	.67
**Asian**	118 (1.9%)	78 (1.3%)	40 (0.6%)	.58
**Black or African American**	582 (9.3%)	425 (6.8%)	157 (2.5%)	<.001
**Native Hawaiian or Other Pacific Islander**	9 (0.1%)	8 (0.1%)	1 (0.0%)	.12
**White**	4526 (72.7%)	2811 (45.1%)	1715 (27.5%)	<.001
**Unknown**	979 (15.7%)	633 (10.2%)	346 (5.6%)	.48
Ethnicity				
**Hispanic or Latino**	2297 (36.9%)	1513 (24.3%)	784 (12.6%)	.006
**Non-Hispanic or Latino**	3000 (48.2%)	1861 (29.9%)	1139 (18.3%)	.01
**Unknown**	930 (14.9%)	590 (9.5%)	340 (5.5%)	.88
Financial class				
**Insured**	5567 (89.4%)	3565 (57.3%)	2002 (32.2%)	.07
**New to UM**	2136 (34.3%)	1320 (21.2%)	816 (13.1%)	.027
**New to BPEI**	3951 (63.4%)	2473 (39.7%)	1478 (23.7%)	.021
**New to Telemedicine**	4178 (67.1%)	2613 (42.0%)	1565 (25.1%)	.009

a Results listed as count (% of total) unless otherwise indicated. Sum of count may be less than the total due to missing data.

b Statistical tests performed are χ^2^ unless otherwise indicated.

c Kruskal-Wallis test utilized to compare medians.

### 3.2 Secondary outcomes

Health insurance information was provided by 5567 (89.4%) patients; the remainder either chose not to disclose or did not have health insurance. Of the 6227 patients seen, 2136 (34.3%) were new to the university-based health system, 3951 (63.4%) were new patients to the eye institute, and 4178 (67.1%) were new to telemedicine. Video conferencing was conducted on 6886 (96.5%) encounters, with only 252 (3.5%) being audio-only encounters.

### 3.3 Patient diagnoses

**Figure 3:**
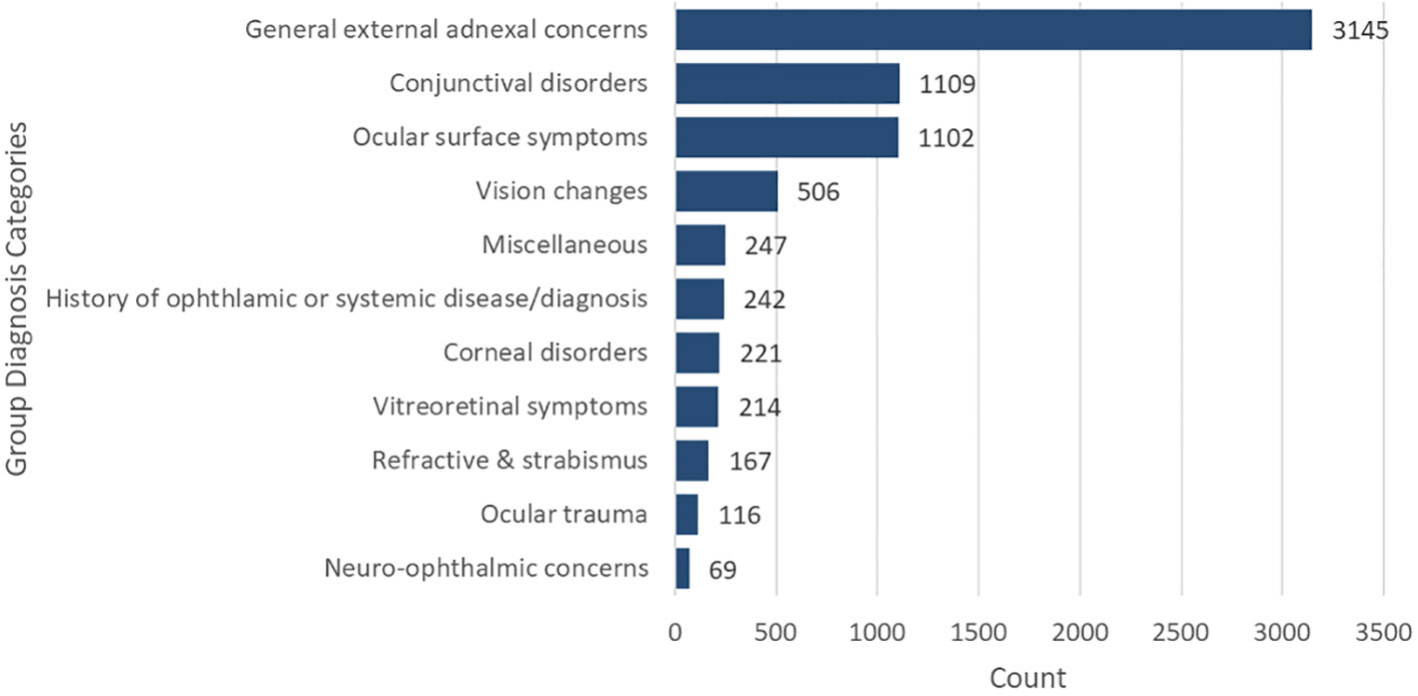
Distribution of primary diagnosis groups.

A review of ICD-10 codes revealed 7138 primary diagnoses associated with the virtual visits. The most common diagnoses were grouped into the category general external adnexal concerns 3145 (44.1%), followed by conjunctival disorders 1109 (15.5%), and ocular surface symptoms 1102 (15.4%) (**Figure 3**).

## 4 Discussion

As a novel virtual program, the purpose of this study was to provide proof-of-principle for patient utilization of a tele-ophthalmology triage program (Rapid Virtual Eye Care^®^) in evaluating acute ocular concerns during the COVID-19 PHE. Over the 31-month period, the on-demand, synchronous patient-to-doctor video conferencing allowed ophthalmologists and optometrists to complete 7138 virtual encounters for 6227 patients, averaging 230 encounters per month; 428 encounters took place in March of 2023, near the end of the PHE.

Utilization of this tele-ophthalmology program increased during the study period. The robust growth in 2021 (**Figure 1**) can be attributed to multiple factors, including establishment of a program leadership position to oversee program details and schedules, dedication of virtual coordinators, technologic advancements to add the self-scheduling feature on the Institute’s website, and a digital marketing campaign to promote the service. Continued oversight of personnel, analysis of different utilization metrics and marketing campaigns remain ongoing. Further, patient and provider perception surveys are analyzed to address concerns to improve the service.

This tele-ophthalmology program was most utilized by women between ages 50-60 years and those of White, non-Hispanic descent with health insurance. These data are corroborated by prior reports; Aziz et al. found men, those who self-identified as Black, those with a high school-level education or less, and patients whose primary language was not English were less likely to seek care via telemedicine (14). Our study did not find a significant difference between male and female tele-ophthalmology use in racial groups with more than 10 patients. Our study demonstrated less use among minority groups. These findings underscore the importance of targeted interventions to increase telemedicine access for underserved communities. Potential strategies include partnering with community leaders, providing digital literacy support, and promoting services through Black-owned media outlets such as local newspapers and national programs like *Roland Martin Unfiltered* (15). Programs designed in collaboration with community organizations, coupled with robust feedback mechanisms, may enhance access to care for socioeconomically disadvantaged populations (15). Education level and primary language were not an outcome measure of this study and further reflection of potential socioeconomic healthcare disparities as it translates to telemedicine is warranted.

Technical and connectivity issues are known barriers to telemedicine (16). Lack of universal access to technical devices and poor internet connection or access varies among populations and locations (16). Despite 67.1% of patients being new to telemedicine, over 96.5% of visits included video conferencing, suggesting technical difficulty connecting to the virtual platform was uncommon in this population. If technological difficulties were encountered with Zoom, our default platform, we attempted connection via Doximity, though we do not have data regarding exact proportions connected via the two platforms. When Doximity was unsuccessful, we converted to audio-only phone encounters for patients who were established with the institute.

Our program provided quick and convenient access to an eye care provider for patients throughout the state of Florida. Tauber et al. previously reported a lack of access to emergency ophthalmic care, especially in rural areas in Florida where 93% of emergency departments do not have an on-call ophthalmologist available; even in the pre-pandemic era, there was significant interest in tele-ophthalmology services for those patients (10, 17). While Bascom Palmer Eye Institute does have an eye emergency room available to those who can travel to Miami, FL, lead times to in-person appointments for non-emergent conditions were long, exceeding 30 days for new patients in most subspecialties due to high demand and limitation of in-person visits. Our unprecedented program provided patients the opportunity to self-schedule their appointment through our online portal within 3 days of their request. We achieved an average lead time under 1.5 days, which allowed patients to discuss their concerns with an eye care provider more quickly. Appointments automatically become available 48-hours in advance via the self-scheduling platform (at 20-minute intervals), and follow-ups can be pre-scheduled internally outside the 48-hour window, when indicated.

In our patient population, external ocular adnexal and ocular surface concerns comprised 75.0% of acute presenting telehealth complaints. The most common diagnoses included eyelid swelling, hordeolum/chalazion, and acute/chronic conjunctivitis which could all be visualized by the eye provider via the video telehealth platform. Similar findings have been reported showing corneal and external disease to be common conditions prompting patients to seek tele-ophthalmology (18). Thus, eye care providers could adequately triage the severity of a patient’s subjective and objective ocular findings and create diagnostic and treatment plans and/or referrals using this model program. Straightforward diagnoses were able to be managed virtually, decreasing the need for in-person visits (10). Managing low-acuity diagnoses virtually is presumably beneficial to both the patient and provider from a time- and cost-efficiency standpoint (3, 19, 20).

While a minority, patients with higher acuity, or less-defined symptoms or findings, were referred for in-person examination and/or testing; examples include patients presenting with acute pain, photophobia, photopsia, new floaters, subjective changes in vision, and diplopia. Physicians were able to make direct subspecialist referrals (cornea, glaucoma, neuro-ophthalmology, retina, etc.) to address patient concerns within the recommended timeframe. By streamlining referrals, we feel the service minimized unnecessary emergency-room visits and expedited in-person visits and treatment and/or surgical interventions. Further review of these referrals is warranted to prove benefit. Evaluation was limited largely to gross external visualization of the globes and external ocular adnexa, gross assessment of extraocular motilities, and presentation of Amsler grid to better elucidate patients presenting with acute metamorphopsia or scotomas.

While our study demonstrated the utility of tele-ophthalmology, its limitations include limited access to data collection between April and September 2020. During this period, Rapid Virtual Eye Care^®^ patients were difficult to identify due to lack of a specific visit type. Much of the published literature report telehealth findings soon after the declaration of the pandemic by the World Health Organization in March 2020. Our data set began 6 months after the start of the pandemic and should be considered when comparing findings. Further evaluation of socioeconomic and access to care disparities are warranted to evaluate why certain populations are less likely to use telehealth as a mode to seek care. Additional investigation into patient follow-up after initial diagnosis and concordance of primary diagnosis with in-person examination will be important to validate this tele-ophthalmology program as a useful adjunct to acute triage care.

The COVID-19 pandemic encouraged the development of new ways to deliver healthcare remotely given the concern for disease exposure during hospital visits. Tele-ophthalmology still plays an important role in the post-PHE era, as multiple studies have shown limitations in access to ophthalmic care in rural and nonrural areas (17). Here, we report the initial outcomes of a novel synchronous, tele-ophthalmology triage program at a U.S. academic center during the PHE. This program was not intended to be a replacement for in-person eye exams, but rather an adjunct triage tool to operate concurrently within a larger ophthalmic institute to improve access and delivery of care. Our study showed access to ophthalmic care for acute ocular concerns could be improved in an academic setting where significant lead times for in-person appointments exist, and for those whose proximity or transportation to clinic may be a barrier. To our knowledge, this is the first tele-ophthalmology triage program of its kind described in the United States, and our preliminary outcomes suggest this model may be generalizable to other states and institutions to improve patient access to care.

## 5 Conclusion

In summary, we feel that synchronous tele-ophthalmology is validated as a beneficial adjunct tool to triage acute patient concerns, especially in the setting of a large academic institution with growing lead times. Our tele-ophthalmology model provided rapid access for patients with acute eye concerns to speak with a licensed eye care physician who could propose an initial treatment/management plan. Further investigations are needed to elucidate the financial benefits or limitations and explore patient outcomes. An overt limitation to growing and perpetuating a remote platform such as this is the looming threat of reduced payor reimbursement.

## Data Availability

The raw data supporting the conclusions of this article will be made available by the authors, without undue reservation.
